# HALP score as a novel prognostic factor for patients with myelodysplastic syndromes

**DOI:** 10.1038/s41598-024-64166-6

**Published:** 2024-06-15

**Authors:** Vildan Gursoy, Sevil Sadri, Hatice Demirci Kucukelyas, Fazıl Cagri Hunutlu, Ibrahim Ethem Pinar, Zafer Serenli Yegen, Nihan Alkış, Tuba Ersal, Ridvan Ali, Vildan Ozkocaman, Fahir Ozkalemkas

**Affiliations:** 1 Department of Hematology, Bursa City Hospital, Bursa, Turkey; 2 Department of Internal Medicine, Bursa City Hospital, Bursa, Turkey; 3 Department of Hematology, Uludag University Medical Faculty, Bursa, Turkey; 4 Department of Hematology, Isparta City Hospital, Isparta, Turkey

**Keywords:** Albumin, Hemoglobin, Lymphocyte, Myelodysplastic syndrome, Platelet, Prognostic factor, Biomarkers, Cancer, Medical research

## Abstract

Myelodysplastic syndrome (MDS) is a heterogeneous spectrum of clonal hematopoietic disorders with varying degrees of cytopenia and morphologic dysplasia. The hemoglobin, albumin, lymphocyte, and platelet (HALP) score is a prognostic marker in several types of malignant tumors. Prognostic value of HALP score remains unclear for MDS. To determine the prognostic value of baseline HALP score in MDS. We retrospectively analyzed data from 130 newly diagnosed MDS patients evaluated and classified under HALP score. By the receiver operating characteristic (ROC) analysis, the optimal cut-off value of HALP was > 67.5 in predicting mortality. Patients were divided into two groups: with low and high HALP scores, and the characteristics were compared between both groups. Patients’ median age was 68 (19–84) years, and 79 (60.8%) were male. Higher HALP score was detected in MDS patients with intermediate-risk under IPSS score, and at high and very high risks under IPSS-R score, and those receiving azacitidine (AZA) treatment. The survival rates of those with a HALP score > 67.5 were significantly lower than those with low HALP score at 17.77 ± 3.98 (median ± SE) (*p* < 0.001). The 3-, 5- and 10-years survival rates of individuals with HALP scores > 67.5 were found as 25, 18, and 11%, respectively. Median overall survival (OS) was also determined as 33.10 (95% CI 16.34–49.88) months by the Kaplan–Meier method. HALP score has shown an ability to be a useful prognostic biomarker in various cancers, including MDS. The meaningful cut-off value of HALP is disease-specific and largely study-specific. High HALP score is associated with unfavorable clinicopathological characteristics. Also, it may be useful in predicting OS and mortality of MDS.

## Introduction

Frequently seen in advanced age, myelodysplastic syndrome (MDS) is a heterogeneous clonal stem cell disease characterized by ineffective hematopoiesis and having a risk of progression to acute myeloid leukemia (AML)^1^. The clinical course of MDS varies considerably, and most patients cannot tolerate intensive treatment approaches due to their advanced age. Accurate diagnosis and reliable prognostic assessment are of critical importance for making individualized clinical decisions in patients with MDS^2^. Therefore, prognostic parameters and/or prognostic scoring systems have been developed to distinguish between patient groups differing in median survival time. An ideal prognostic scoring system should contain merely independent prognostic parameters, and the limits of these parameters should be validated, easily identifiable at diagnosis, and cost-effective. The international prognostic scoring system (IPSS), the revised IPSS (IPSS-R), and the World Health Organization (WHO) prognostic scoring system [WPSS] are the most commonly utilized prognostic models^3,4^. However, it is considered that many factors, except for the variables in these systems, may also affect the prognosis of MDS^5^.

Immune response and inflammatory cells have been defined as very important parameters in cancer survival rates^6^. In several recent studies, peripheral blood cells, including absolute lymphocyte count (ALC), monocytes, and platelets (Plt), have been reported to be related to higher mortality rates in lymphoma patients^7,8^. In recent years, many combinations of inflammatory parameters, such as neutrophil–lymphocyte ratio, platelet-lymphocyte ratio, and lymphocyte-monocyte ratio have been used to predict the prognosis in patients with oncological and hematological malignancies^9–11^. Nutritional status has also been emphasized in various studies as an important factor affecting the prognosis^11–13^. Previous studies have defined a new inflammatory index called the hemoglobin, albumin, lymphocyte, and platelet (HALP) score, which is effective in the prognosis of those with kidney, bladder, stomach, small-cell lung, and prostate cancers^14–17^. The HALP score is calculated using four laboratory markers (hemoglobin, albumin, lymphocyte, and platelet), which are the biomarkers of nutrition and inflammation status. In the field of hematology, the prognostic efficiency of the HALP score has been evaluated in a limited number of studies, including in diffuse large B-cell lymphoma and multiple myeloma^18,19^. Based on the literature, however, there are no studies evaluating the relationship between the HALP score and the prognosis of MDS patients. Therefore, our study aims to investigate the role of the HALP score, as well as the IPSS and IPSS-R scoring systems, in the prognosis and overall survival (OS) of MDS patients before the treatment.

## Materıals and methods

### Study design and patients’ selection

The data of 130 patients with newly diagnosed MDS between January 2010 and March 2023 were analyzed retrospectively in our study. Diagnosed with MDS in the adult hematology outpatient clinics of Bursa Uludag University Hospital and Bursa City Hospital, patients (≥ 18 years) having all examinations at the time of diagnosis, without nephrotic level proteinuria, and complying with the follow-ups regularly, and not having a second malignancy were included in the study. Those with an unclear diagnosis of MDS based on bone marrow biopsy, having additional non-MDS hematological diseases or solid organ malignancies with ongoing treatment, not complying with follow-ups, and those whose hospital records could not be accessed were not included in the study. The demographic data, clinicopathological features, laboratory findings, comorbidities, treatment regimes, responses to the treatment, transfusion frequency, and OS rates of the patients were evaluated using the data from the MIA-Med patient registry system and obtained from the FONET software program (version 3.1.1.6 b5). The cases were also grouped according to the IPSS, IPSS-R, and HALP scores. The primary endpoint of our study was to evaluate and classify newly diagnosed MDS patients according to the HALP score. Under the receiver operating characteristic curve (ROC) analysis, the optimal cut-off value of the HALP was > 67.5 units to predict mortality. Even so, the secondary endpoint was to show any relationship between the HALP score, and other prognostic factors, such as mortality and OS.

### The hemoglobin, albumin, lymphocyte, and platelet score (HALP)

In the literature, the hemoglobin, albumin, lymphocyte, and platelet (HALP) score is calculated with the following formula: Hemoglobin (g/L) × albumin (g/L) × lymphocytes (/L) /platelets (/L)^15,20^. In our study, the HALP score was calculated from the laboratory data obtained within the first 10 days since the diagnosis, and the pre-treatment data were used. The values in the follow-up period were not used for the calculation of the HALP score.

### Statistical approach

The normality of numerical data distribution was assessed using the Shapiro–Wilk test. The numerical variables with normal distribution were presented as mean ± standard deviation (SD) and compared between the groups using the student *t*‑test or the one‑way analysis of variance (ANOVA). The non-normally distributed numerical variables were presented as the median and interquartile range (IQR [Q1–Q3]) and compared between the groups using the non-parametric Mann–Whitney U test or Kruskal–Wallis ANOVA tests. The categorical variables were also expressed as frequencies and compared using Fisher’s exact test or the chi‑square test where suitable. The IPSS, IPSS-R, and HALP scores were compared with Spearman’s rank coefficient of correlation. The cut-off point of the HALP scores was determined to estimate the mortality. The optimal cut-off value of the HALP score was defined as the point on the ROC curve closest to the 0% false-positive and 100% true-positive marks. The survival curves of all 130 patients were analyzed using the Kaplan–Meier and log-rank tests. The Statistical Package for Social Sciences (SPSS) software for Windows 20.0 (SPSS, IBM Inc., Chicago, USA) was used for all statistical analyses, and statistical significance was established at the *p*-value of < 0.05.

### Ethical approval

All procedures in the present study were performed under the ethical standards of the institutional and/or national research committee and with the 1964 Helsinki Declaration and its later amendments or comparable ethical standards. This study was approved by the local ethical committee (Uludag University Faculty of Medicine, Bursa-2023-7/25, Date: 11th April 2023). Informed consent was also obtained from all individual participants in the study.

## Results

The performance of the HALP score in predicting mortality was evaluated based on the ROC curves. Based on the ROC analysis, the optimal cut-off value of HALP was > 67.5 units. The area under the curve (AUC) was detected as 0.639 (95% CI 0.542–0.736), and the *p*-value was 0.006 with a sensitivity of 50% [95% confidence interval (CI) 45.1–76.2] and a specificity of 77.3% (95% CI 60.4–83.2).

### Patients’ characteristics

A total of 130 patients with newly diagnosed MDS were included in the analyses. Of 130 cases, the median age was 68 (19–84) years, and 79 (60.8%) were male. The characteristics of the cases by the HALP score are summarized in Table 1. In terms of the IPSS, IPSS-R risk groups, and use of erythropoietin (EPO) and AZA by the cases, a statistically significant difference was observed, compared with the HALP score (*p* < 0.05). While the number of patients with a HALP score > 67.5 was found higher in MDS patients with intermediate-risk under the IPSS score, with high and very high risks by the R-IPSS score and receiving AZA treatment, the number of those with the HALP score ≤ 67.5 was found to be higher among the cases treated with EPO.

**Table 1:** Comparisons of demographic and clinical characteristics of the cases in HALP score groups.

	HALP score ≤ 67.5	HALP score > 67.5	Total	*p*
	n (%)/M [Q1–Q3]	n (%)/M [Q1–Q3]	n (%)/M [Q1–Q3]	
Sex				
Male	48 (57.8)	31 (66)	79 (60.8)	0.362
Female	35 (42.2)	16 (34)	51 (39.2)	
IPSS				
Low	32 (38.6)	7 (14.9)	39 (30)	**0.006**
Intermediate	46 (55.4)	39 (83)	85 (65.4)	
High	5 (6)	1 (2.1)	6 (4.6)	
R-IPSS				
Very low-risk MDS	6 (7.2)	4 (8.5)	10 (7.7)	**0.004**
Low-risk	41 (49.4)	10 (21.3)	51 (39.2)	
Intermediate-risk	19 (22.9)	10 (21.3)	29 (22.3)	
High-risk	11 (13.3)	18 (38.3)	29 (22.3)	
Very high-risk	6 (7.2)	5 (10.6)	11 (8.5)	
Ring sideroblasts in BM				
Yes	29 (34.9)	17 (36.2)	46 (35.4)	0.888
No	54 (65.1)	30 (63.8)	84 (64.6)	
Use of EPO				
Yes	50 (60.2)	16 (34)	66 (50.8)	0**.004**
No	33 (39.8)	31 (66)	64 (49.2)	
Transformation to AML				
Yes	10 (12)	6 (12.8)	16 (12.3)	0.905
No	73 (88)	41 (87.2)	114 (87.7)	
Use of AZA	0 (0)			
Yes	26 (31.3)	30 (63.8)	56 (43.1)	** < 0.001**
No	57 (68.7)	17 (36.2)	74 (56.9)	
Use of decitabine				
Yes	5 (6)	1 (2.1)	6 (4.6)	0.417
No	78 (94)	46 (97.9)	124 (95.4)	
Allo-BMT	0 (0)			
Yes	1 (1.2)	1 (2.1)	2 (1.5)	0.681
No	82 (98.8)	46 (97.9)	128 (98.5)	
Use of venetoclax				
Yes	6 (7.3)	3 (6.4)	9 (7)	0.841
No	76 (92.7)	44 (93.6)	120 (93)	
Chelation therapy				
Yes	25 (30.1)	9 (19.1)	34 (26.2)	0.171
No	58 (69.9)	38 (80.9)	96 (73.8)	

The *p*-value was obtained from the chi-square or Fisher’s Exact test. Allo-BMT: Allogenic bone marrow transplantation, *AML* Acute myeloid leukemia, *AZA* Azacitidine, *BM* Bone marrow, *EPO* Erythropoietin, *HALP Score* Hemoglobin, albumin, lymphocytes, and platelet score, *IPSS-R* Revised international prognostic scoring system, *MDS* Myelodysplastic syndrome, M: Median[Q1–Q3].

Significant values are in [bold].

### Association between the HALP score and quantitative clinical outcomes

In the present study, the distribution of socio-demographic and clinical characteristics of the patients with HALP scores ≤ 67.5 and > 67.5 were examined, and the findings are given in Table 2. Accordingly, given the subsets of the HALP score, a statistically significant difference was seen in the blasts of bone marrow (BM) (%), leukocytes, lymphocytes, neutrophils, platelet (Plt), lactic dehydrogenase (LDH), IRSS-R score, AZA chemotherapy (AZA Chemo) count, transfusion counts of erythrocyte suspension (ES) and thrombocyte suspension (TS) within the last year (*p* < 0.05). Among the cases with the HALP score > 67.5, the findings were determined to be as follows: the blasts of BM 5%, [1–10]; leukocytes, 4900 [3560–7990]; lymphocytes, 1756 [1270–2696], neutrophils, 2410 [1390–4340]; Plt, 36,400 [28400–74400], LDH, 245 [177–359]; IPSS-R score, 4.5 [3–6]; AZA Chemo count, 2 [0–6]; thrombocyte value, 13 000 [0–29]; last-year ES, 10 [2–22], and TS transfusion, 6 [0–20]. Even so, a significant difference was observed among the cases with the HALP scores ≤ 67.5 (*p* < 0.05).

**Table 2:** Comparisons of quantitative clinical features of the cases in HALP score groups.

	Total	HALP score ≤ 67.5	HALP score > 67.5	*p*
	M [Q1–Q3]	M [Q1–Q3]	M [Q1–Q3]	
Disease duration (month)	15.13 [7.37–35.47]	21.73 [7.77–54.13]	12.3 [7.17–24.03]	0.093
Diagnostic age	68 [60–75]	68 [60–76]	69 [60–73]	0.810
Age	70 [66–78]	71 [67–79]	70 [65–77]	0.696
Blasts in BM (%)	2 [1–8]	1 [0–6]	5 [1–10]	**0.005**
Leukocyte	4010 [2760–6110]	3620 [2540–5770]	4900 [3560–7990]	**0.007**
Neutrophil	2000 [1090–3270]	1753 [990–2990]	2410 [1390–4340]	**0.042**
Lymphocyte	1365 [1030–1890]	1240 [860–1600]	1756 [1270–2696]	** < 0.001**
Hgb	8.52 [7.5–9.79]	8.3 [7.3–9.47]	8.8 [7.6–10.1]	0.061
Hct	25 [22–29.4]	24.9 [21.9–28]	26.6 [23–30.9]	0.070
Plt	116,000 [47000–239300]	188,100 [116000–299000]	36,400 [28400–74400]	** < 0.001**
TP	69 [64–74]	69 [64–72]	70 [64–76]	0.221
Albumin	40 [36–43]	41 [35–43]	39 [36–43]	0.478
LDH (IU/L)	217 [169–292]	207 [165–275]	245 [177–359]	**0.014**
Ferritin	462.5 [166–900]	441 [168–900]	541 [156–902]	0.601
R-IPSS	3.5 [2.5–5]	2.5 [2–4.5]	4.5 [3–6]	**0.001**
AZA chemo count	0 [0–4]	0 [0–2]	2 [0–6]	** < 0.001**
ES transfusion count	12 [3–31]	11 [2–28]	16 [5–35]	0.262
Thrombocyte transfusion count	6 [0–17]	3 [0–13]	13 [0–29]	**0.017**
Last-year ES transfusion count	4 [0–16]	1 [0–12]	10 [2–22]	**0.001**
Last-year TS transfusion count	0 [0–11]	0 [0–6]	6 [0–20]	** < 0.001**
Ferritin at diagnosis	448 [166–902]	441 [168–928]	470 [156–902]	0.704
Ferritin at last follow-up	1379 [382–2882]	922 [286–2937]	1928 [432–2787]	0.153

The *p*-value was obtained with the Mann–Whitney U test. *AZA Chemo* Azacitidine chemotherapy, *ES* Erythrocyte suspension, *HALP Score* Hemoglobin, albumin, lymphocytes, and platelet score, *Hct* Hematocrit, *Hgb* Hemoglobin, *IPSS-R* Revised international prognostic scoring system, *LDH* Lactate dehydrogenase, *M* Median, [Q1–Q3], *Plt* Platelet, *TP* Total protein, *TS* Thrombocyte suspension.

Significant values are in [bold].

### Univariate and multivariable analyses for overall survival

After the univariate logistic regression (LR) analysis, such features as sex, the scores of HALP and IPSS, treatment with EPO and AZA, transformation to AML, blasts in BM (%), Plt, LDH, IPSS-R, AZA Chemo count, ES, and thrombocyte transfusions, the increase in the frequency of last-year ES and TS transfusions were observed to be statistically significant risk factors having effects on the mortality of the cases (*p* < 0.05). In addition, the value of albumin was found to be a variable having a protective effect on mortality, [OR: 0.87 (95% CI 0.82–0.94)]. The use of EPO was also detected to be a protective factor against the mortality in the cases [OR: 0.17 (95% CI 0.08–0.36)].

Compared to the cases with a HALP score ≤ 67.5, those with HALP scores > 67.5 were seen to have 3.40 times (95% CI 1.60–7.24) higher mortality risk (*p* = 0.002). Even so, the cases with intermediate IPSS scores were found to have a mortality risk of 3.76 times (95% CI 1.63–8.69) higher, compared to the cases with low IPSS scores (*p* = 0.002). The rates of encountering the transformation to AML were 19.9-fold (95% CI 2.54–155.80) higher among the patients, and 6.06-fold (95% CI 2.81–13.08) higher in those receiving AZA. In addition, while the cases with increased blasts in BM (%) had a 1.22 times (95% CI 1.11–1.33) risk of mortality, the risk was found to be 1.02 times higher (95% CI 1.01–1.08) and 3.33 times (95% CI 1.62–6.84) higher in those with increased thrombocyte replacement and LDH, respectively. It was determined, however, that the increase in the IPSS-R score had a 1.64-fold (95% CI 1.31–2.07) higher mortality risk (*p* < 0.05).

In the stepwise multivariate LR (Enter method) model established with the variables where the significance was observed as a result of the univariate LR analysis, the HALP score and transformation to AML were observed to be the risk factors having a significant effect on the mortality (*p* > 0.05). It was also observed that the protective effect of the albumin variable continued in the multivariate LR model (*p* = 0.001). In the Stepwise Multivariate LR model, established by adding the age and gender to the variables whose significance was observed as a result of the Univariate LR analysis, such factors as age, transformation to AML, albumin, number of ES transfusions were observed as risk factors having significant effects on mortality (*p* < 0.05) (Table 3).

**Table 3:** Univariate and multivariate logistic regression analysis of prognostic factors.

	Univariate LR		Multivariate LR	
	OR (95% CI)	*p*	OR (95% CI)	*p*
HALP > 67.5	3.40 (1.60–7.24)	**0.002**	3.09 (0.67–14.16)	0.147
Age > 70	1.58 (0.78–3.19)	0.203	3.83 (1.05–14.01)	**0.042**
Sex (Male)	1.31 (0.65–2.66)	0.449	2.21 (0.63–7.35)	0.197
IPSS Low	1	**0.008**	1	0.775
Intermadiate	3.76 (1.63–8.69)	**0.002**	1.79 (0.36–8.78)	0.475
High	4684.01 (0–54,456.15)	0.990	27,501.12 (0–65,461.45)	0.999
Ring sideroblast in BM (1)	0.55 (0.27–1.15)	0.112		
EPO (1)	0.17 (0.08–0.36)	** < 0.001**	0.49 (0.78–3.112)	0.451
Transformation to AML (1)	19.90 (2.54–155.80)	**0.004**	23.37 (1.49–364.75)	**0.025**
AZA (1)	6.06 (2.81–13.08)	** < 0.001**	1.41 (0.14–13.86)	0.767
Decitabine (1)	2.13 (0.38–12.08)	0.392		
Allogenic (1)	1.03 (0.06–16.85)	0.983		
Venetoclax (1)	1.29 (0.33–5.05)	0.712		
Chelation (1)	0.76 (0.35–1.66)	0.488		
Blasts in BM (%)	1.22 (1.11–1.33)	** < 0.001**	1.16 (0.95–1.41)	0.134
Leukocyte	1.01 (0.09–1.19)	0.102		
Neutrophil	1.01 (0.90–1.25)	0.201		
Lymphocyte	1 (0.50–1.25)	0.576		
Hgb	0.89 (0.73–1.10)	0.297		
Hct	0.96 (0.90–1.03)	0.230		
Plt	3.33 (1.62–6.84)	**0.001**	2.01 (1–1.54)	0.115
TP	0.97 (0.92–1.01)	0.162		
Albumin	0.87 (0.82–0.94)	** < 0.001**	0.81 (0.72–0.92)	**0.001**
LDH (IU/L)	1.02 (1.01–1.08)	**0.025**	1.01 (0.99–1.02)	0.133
Ferritin	1.13 (0.57–2.25)	0.726		
IPSS-R	1.64 (1.31–2.07)	** < 0.001**	0.68 (0.38–1.21)	0.733
AZA chemo count	1.11 (1.01–1.21)	**0.027**	0.88 (0.76–1.02)	0.052
ES Transfusion count	1.02 (1.01–1.03)	**0.007**	1.03 (1.01–1.05)	0.024
Thrombocyte count	1.06 (1.03–1.09)	** < 0.001**	0.99 (0.93–1.08)	0.988
Last-year ES transfusion count	1.11 (1.06–1.17)	** < 0.001**	1.03 (0.96–1.10)	0.424
Last-year thrombocyte transfusion count	1.11 (1.05–1.19)	** < 0.001**	1.01 (0.91–1.13)	0.831

Bold values are statistically significant (*p* < 0.05) (1) and mean that it exists. *AZA Chemo* Azacitidine chemotherapy, *CI* Confidence interval, *ES* Erythrocyte suspension, *HALP Score* Hemoglobin, albumin, lymphocytes, and platelet score, Hct: Hematocrit, *Hgb* Hemoglobin, *IPSS-R* Revised international prognostic scoring system, *LDH* Lactate dehydrogenase, *LR* Logistic regression. *OR* Odds ratio, *Plt* Platelet, *TP* Total protein, *TS* Thrombocyte suspension.

Significant values are in [bold].

The Spearman’s rank coefficient of correlation was calculated to determine the association between the IPSS, IPSS-R, and HALP scores. There was a moderately positive correlation between the IPSS, IPSS-R, and HALP scores (respectively r = 0.303, r = 0.368; *p* < 0.001). Higher IPSS or IPSS-R results in higher HALP scores (Table 4).

**Table 4:** Comparison between IPSS, IPSS-R, and HALP score.

		HALP score	IPSS
IPSS	r	0.303	
	*p*	< 0.001	
R-IPSS	r	0.368	0.721
	*p*	< 0.001	< 0.001

r: The Spearman’s rank coefficient (n = 130).

**Figure 1:**
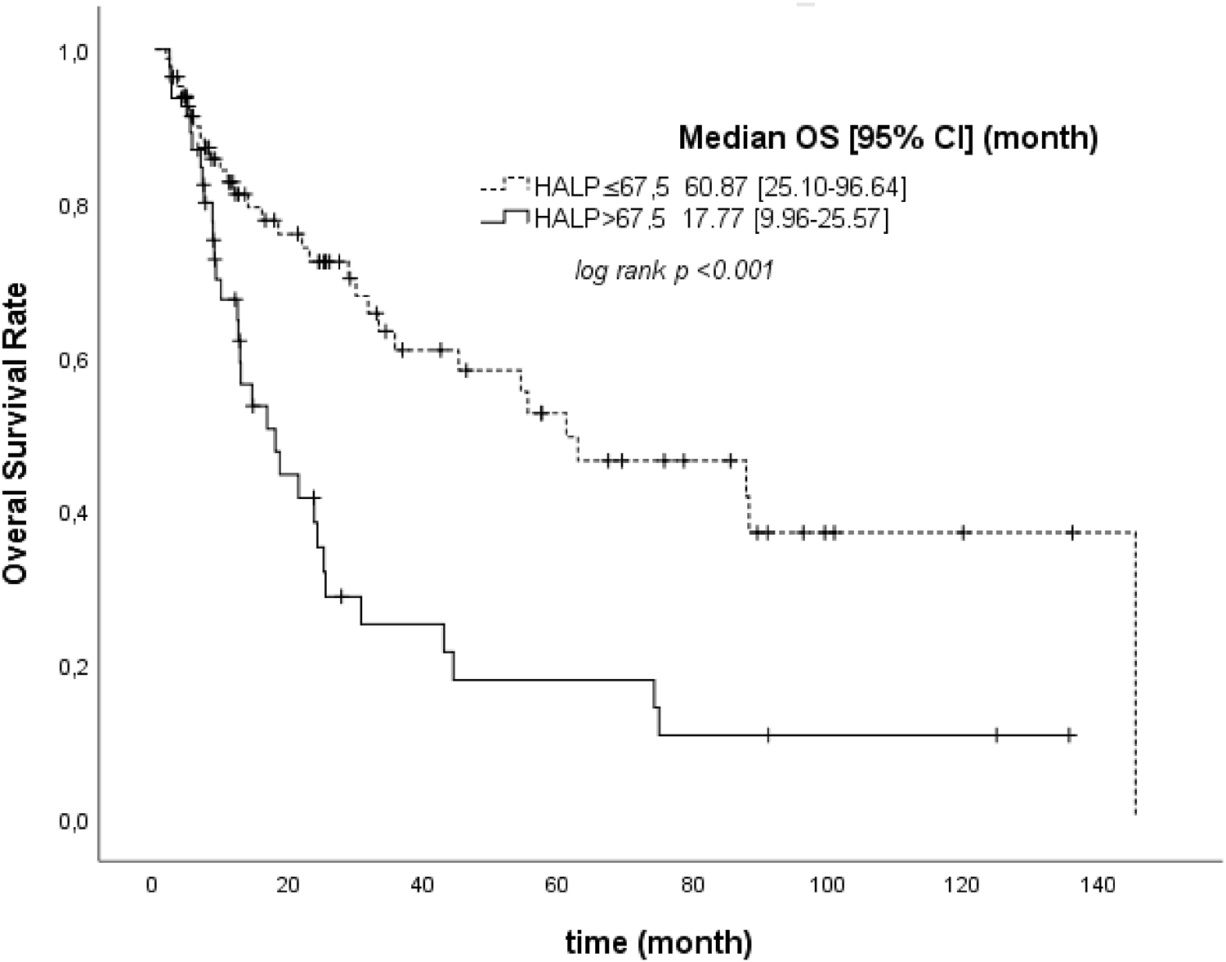
The Kaplan–Meier curves of overall survival (OS) for HALP.

**Figure 2:**
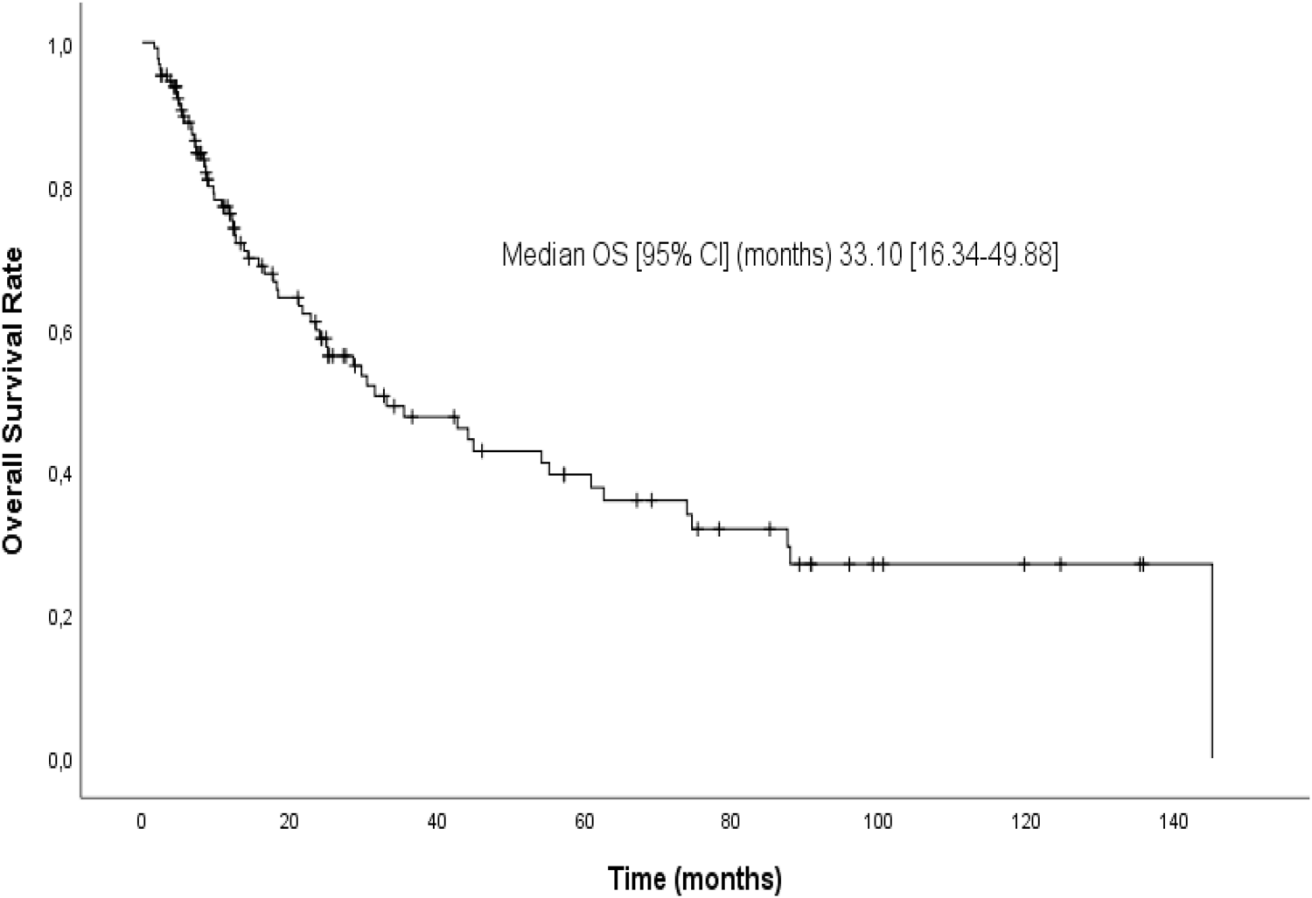
The Kaplan–Meier curve of overall survival (OS) for HALP.

The survival time of the individuals with a HALP score > 67.5 was seen to be significantly lower as the median ± SE (17.77 ± 3.98), compared to the cases with a HALP score ≤ 67.5 (*p* < 0.001) (Fig. 1). Given the findings of the Kaplan–Meier test, the 3-, 5-, and 10-years year survival rates of the individuals with the HALP score > 67.5 were calculated as 25, 18, and 11%, respectively (Table 5). However, the median OS was calculated as 33.10 (95% CI 16.34–49.88) based on the Kaplan–Meier method (Fig. 2).

**Table 5:** The Kaplan–Meier survival values of the cases in HALP > 67.5 and HALP ≤ 67.5 groups and comparisons with the Log-rank test.

	Exitus	Survival	Estimated survival	Estimated survival rates in 3rd, 5th and 10th years	p*
	n (%)	n (%)	median ± SE		
HALP					
≤ 67.5	32 (38.6)	51 (61.4)	60.87 ± 18.25	0.61 / 0.50 / 0.37	** < 0.001**
> 67.5	32 (68.1)	15 (31.9)	17.77 ± 3.98	0.25 / 0.18 / 0.11	

The Kaplan–Meier Test, *Log-rank (Mantel-Cox), *HALP Score* Hemoglobin, albumin, lymphocytes, and platelet score, *SE* Standard error.

Significant values are in [bold].

## Discussion

In the present study, by analyzing 130 patients with MDS retrospectively, we revealed that the HALP score can be a novel prognostic index that can be easily evaluated, as well as well-known prognostic indices, such as WPSS, IPSS, and IPSS-R, in the diagnosis of MDS. We determined the HALP score before the treatment period, compared survival rates between the groups with low and high HALP scores (≤ 67.5 and > 67.5), and found that high HALP scores were strongly associated with poor outcomes in MDS patients.

HALP is an immune nutritional biomarker used to predict the prognosis in patients with malignancy. Developed by Chen et al. to predict the prognosis in gastric cancer, the HALP score is calculated through the following formula: [hemoglobin (g/L) × albumin (g/L) × lymphocytes (/L)]/platelets (/L)^15^. These four markers are essential components for predicting the immune and nutritional status of cancer patients. Anemia is a well-documented phenomenon developing in patients with cancer and may occur through several mechanisms like anemia of chronic diseases, anemia of inflammation due to pro-inflammatory cytokines such as IL-6, suppression of erythropoiesis, iron deficiency anemia due to the losses from the gastrointestinal tract^21,22^. The levels of albumin are influenced by a patient’s nutritional state and metabolic demands, and inflammation and high nutritional risks have both been linked to low albumin levels^23^. During an inflammatory process, it is noted that albumin levels decrease while C-reactive protein (CRP) tends to rise. Therefore, albumin levels are a well-known indicator of the prognosis of various cancers^24,25^. Lymphocytes are significant in immunosurveillance, assisting tumor detection and destruction; thus, the depletion of lymphocyte count is considered to play an important role in the prognosis of cancer^26^. Thrombocytes are known to play a part in the metastatic abilities of cancers^27^. By releasing vascular endothelial growth factor (VEGF), thrombocytes promote tumor angiogenesis, along with other inflammatory mediators^28,29^. Additionally, thrombocytes have also been demonstrated to have a role in protecting tumor cells from immune detection^30^. Accordingly, pro-inflammatory conditions may result in thrombocytosis, and reactive thrombocytosis can be observed in solid organ malignancies. However, in our patient group, thrombocytopenia, anemia, and leukopenia are anticipated rather than thrombocytosis due to morphological dysplasia in hematopoietic cells in BM, a decrease in the production of thrombocytes, and ineffective hematopoiesis.

The prognosis of MDS is primarily affected by disease-related parameters, such as low blood cell counts. Especially neutropenia and thrombocytopenia are the major causes of MDS-related deaths. Due to the heterogeneity of clinical manifestations, the survival rate of patients ranges from approximately a few months to several years. While the patients in the low-risk prognostic group have almost the same life expectancy as the age- and sex-adjusted non-MDS population, the survival rate may decrease by less than 1 year in high-risk patients with leukemic transformation and resistance to treatment. Accurately predicting the prognosis of patients at the time of diagnosis may be beneficial so that MDS can be managed successfully, and patients have timely access to the appropriate treatment options. The treatment modalities should aim to improve patients’ well-being and life expectancy. While such options as EPO and supportive treatments are chosen in low-risk MDS patients, other options such as treatments with AZA, decitabine, and venetoclax, intensive induction treatments, and allogeneic stem cell transplantation may be preferred for high-risk MDS patients. Lenalidomide has major efficacy in low-risk MDS with deletion 5q, and iron chelation can be considered when multiple red blood cell transfusions are required. The role of new drugs, including venetoclax or, in case of specific mutations, isocitrate dehydrogenase-1 (IDH1) or IDH2 inhibitors, is investigated. Several prognostic scoring systems have now been developed to classify the risk of MDS patients^31^. These systems are evaluated based on various variables such as blast percentage in BM, genetics, and laboratory tests. However, although such scoring systems display good performance in predicting the prognosis of patients with MDS, the classification of the patients can be carried out based on the findings obtained from repeated outpatient evaluations. In our study, it was seen that the HALP score could be obtained by calculating the laboratory parameters in an inexpensive and easily accessible way at the first outpatient visit, and HALP was also found to be useful in predicting the prognosis. In addition, because of evaluating inflammation and nutritional status, MDS can be utilized as a complement to other scoring systems that have proven effectiveness.

HALP appears to be a valuable prognostic indicator mainly of OS across various cancer subtypes^32–35^. In a study where 1332 patients diagnosed with gastric carcinoma were retrospectively examined by Chen et al. in terms of the prognostic value of the HALP score, the cut-off value of the HALP score was determined as 56.8, and while patients with low HALP scores were found to have a 3-years cancer-specific survival rate of 59.7%, those with high HALP scores were seen to have a score of 74.7%^15^. In another study, the low HALP score (cut-off, 38.8) was also found as a marker of bad prognosis in 834 oesophageal squamous cell carcinoma patients^32^. In a 2021 study in which Vlatka et al. retrospectively queried 153 newly diagnosed diffuse large B-cell lymphoma patients receiving the R-CHOP therapy of rituximab, cyclophosphamide, doxorubicin, vincristine, and prednisone, the authors revealed that a lower HALP score (cut-off, 20.8) was associated with unfavorable clinicopathological characteristics and a predictor of long-term survival; the diffuse large B-cell lymphoma patients with Low HALP were also found to have significantly less 5-years survival (47.3% vs. 79.5%, *p* < 0.001)^18^. However, in the study where Solmaz et al. evaluated the HALP score in 200 patients with multiple myeloma, the cut-off value was found to be 28.8, and a shorter OS was observed in patients with low HALP scores^19^. Different from the findings reported in the literature, we found the cut-off value of the HALP score as 67.5 through the ROC curve analysis in our study. Contrary to previous studies reporting that a high HALP score is a positive indicator of prognosis in solid and hematological malignancies, high HALP scores were found to be associated with poor prognosis in MDS patients in our study^18,19,35–38^. As regards this feature, the depth of thrombocytopenia can be asserted to be the most important determinant between high HALP scores and poor prognosis in MDS. In our study, univariate and multivariate analyses were also performed to investigate the prognostic factors affecting the progression of the disease and deaths. Our findings reveal that the HALP score is a prognostic component for MDS patients. In our study, the 3-, 5-, and 10-years survival rates of MDS patients with a HALP score above 67.5% were calculated as 25, 18, and 11%, respectively. Therefore, validation studies investigating the optimal cut-off point of the HALP score are needed.

In conclusion, HALP has shown an ability to be a useful prognostic biomarker in various cancers, including MDS. There is no standardized or universal threshold of the HALP score to stratify the risks of these outcomes. Based on the extant literature, the meaningful cut-off value of HALP is disease-specific and largely study-specific. In our study, it can be said that a high HALP score is an important predictor of mortality, along with other prognostic factors in the follow-up of MDS patients. Our findings are limited by two‑center experience, retrospective study design, and small sample size restraining the statistical power for some of the presented analyses. More studies are needed in the future to determine optimal cutoff values for the Halp score in this specific patient population. In addition, potential confounding factors could influence the HALP score, including comorbid conditions, the treatments received before the calculation of the HALP score, and demographic factors. A single collection of the lab values may not be enough to fully understand the clinical implications. While it may have potential as a prognostic marker, the HALP score cannot replace the established indices (such as cytogenetics) for MDS. We consider that further external validations are needed to elucidate the accurate prognostic role of the HALP score for MDS patients.

## Data Availability

The datasets used and/or analyzed during the current study are available from the corresponding author upon reasonable request.
